# Assessing Motor Performance and Ankle Mobility in Pre-Adolescent Male Fencers

**DOI:** 10.3390/life15060942

**Published:** 2025-06-12

**Authors:** Yalcin Aydin, Gurkan Tokgoz, Nurkan Yilmaz, Ilgin Ali Coskun, Aysegul Beykumul, Enes Colak, Caner Aygoren, Samet Koc, Fahri Safa Cinarli

**Affiliations:** 1 Faculty of Health Sciences, Malatya Turgut Ozal University, Malatya 44210, Türkiye; yalcin.aydin@ozal.edu.tr; 2 Faculty of Sport Science, Munzur University, Tunceli 62000, Türkiye; gurkantokgoz@munzur.edu.tr; 3 Faculty of Sport Science, Inonu University, Malatya 44000, Türkiye; nurkan.yilmaz@inonu.edu.tr (N.Y.);; 4 Physical Medicine and Rehabilitation Department, Gazi University, Ankara 06560, Türkiye; abeykumul@yahoo.com; 5 Department of Exercise and Sport Sciences, Malatya Turgut Ozal University, Malatya 44210, Türkiye; enescolak4405@gmail.com; 6 Health Sciences Institute, Munzur University, Tunceli 62000, Türkiye; canerkarate4423@gmail.com; 7 Provincial Directorate of Youth and Sports, Malatya 44070, Türkiye; samet_koc87@hotmail.com

**Keywords:** fencing, joint range of motion, functional performance, youth athletes

## Abstract

Ankle mobility plays a critical role in dynamic stability and propulsion during fencing-specific actions. However, its relationship to performance metrics in young athletes remains unclear. This study aimed to investigate the relationship between ankle range of motion (ROM), acceleration, and jump height in pre-adolescent male fencers, as well as to examine bilateral differences in ankle mobility between the front and rear foot. Fifteen fencers (age 10.86 ± 0.91 years) were assessed for ankle ROM (dorsiflexion, plantar flexion, inversion, eversion) using a goniometer. Performance tests included a 7 m sprint lunge (acceleration) and countermovement jump. Significantly large correlations were observed between front and rear foot ankle plantar flexion range of motion and both acceleration (*r* = 0.625–0.628, *p* < 0.05) as well as vertical jump height (*r* = 0.579–0.647, *p* < 0.05). Rear foot ankle plantar flexion range of motion significantly predicted acceleration (*r*^2^ = 0.335, *p* < 0.05) and jump height (*r*^2^ = 0.418, *p* < 0.05). In contrast, no meaningful associations were found between dorsiflexion, inversion, or eversion range of motion and performance metrics. Additionally, bilateral comparisons revealed significantly greater dorsiflexion in the front foot ankle and greater eversion in the rear foot ankle (*p* < 0.05). Plantar flexion ROM is a key contributor to acceleration and jump capacity in youth fencers. Incorporating ankle mobility training may support physical development in this population.

## 1. Introduction

Fencing is a dynamic, multidimensional sport that uniquely combines physical prowess, strategic decision-making, and technical precision [1,2]. Governed by distinct rules for its three primary weapons (foil, sabre, and épée), fencing demands rapid bursts of activity interspersed with periods of rest, typifying its intermittent and high-intensity nature [3,4,5]. Athletically, fencing performance relies on high-intensity actions characterized by rapid force production, swift directional changes, and precise motor coordination, driven by both anaerobic and aerobic energy systems [6,7].

Despite these multifaceted demands, research on fencing biomechanics has primarily concentrated on joint mechanics associated with key movements such as lunges and fleches, while comparatively little attention has been paid to the ankle joint [8,9]. Notably, elite fencers have been shown to exhibit greater ankle extension during lunges [10], underscoring the functional contribution of this joint to fencing-specific performance. The ankle plays a critical role in maintaining stability, facilitating rapid directional transitions, and generating propulsive force—functions that are largely driven by the plantar flexors during high-velocity, fencing-specific actions such as lunges and fleches [11]. However, the influence of specific ankle range of motion components, such as plantar flexion during push-off or dorsiflexion during landing phases, on fencing performance remains insufficiently defined in the available literature.

Although some studies have reported positive associations between ankle dorsiflexion range of motion and high-velocity lower-limb movements such as jumping and sprinting [12], this evidence largely stems from adult or diverse athletic populations. Existing research has predominantly focused on lower-limb strength and power, with limited attention to joint mobility, particularly in the context of youth fencing [13,14]. For example, preliminary evidence suggests that ankle mobility may influence movement velocity during fencing-specific actions, such as the lunge [15]. However, such relationships remain underexplored in pre-adolescent populations. Given the ongoing neuromuscular and structural maturation in pre-adolescent athletes, it is essential to explore whether these biomechanical parameters hold predictive value for performance at early stages of development.

At this stage of development, mobility not only contributes to mechanical efficiency but also plays a fundamental role in motor control processes, including postural stability, bilateral coordination, and proprioceptive regulation [16]. These neuromuscular functions are essential during childhood and early adolescence, a period characterized by rapid changes in motor organization and sensorimotor integration [17]. These developmental aspects highlight the broader significance of ankle mobility beyond pure performance outcomes.

To the best of our knowledge, this is the first study to examine the relationship between ankle range of motion and fencing performance metrics, namely vertical jump height and acceleration derived from a fencing-specific 7 m sprint test, in pre-adolescent male fencers. By addressing this knowledge gap, the study aims to support early-stage training strategies for young fencers by identifying biomechanical markers that may assist in motor development, foundational movement efficiency, and performance-related physical parameters relevant to fencing. This study aimed to investigate the relationship between ankle range of motion, acceleration, and vertical jump height in pre-adolescent male fencers, as well as to compare bilateral ankle mobility between the front and rear foot. It is hypothesized, based on prior research [18,19], that ankle range of motion would demonstrate significant correlations with both jump performance and acceleration performance. Additionally, it was expected that bilateral differences in ankle mobility would be observed between the front and rear foot due to the asymmetrical demands of fencing.

## 2. Materials and Methods

### 2.1. Study Design and Participants

This study was designed as a cross-sectional investigation adhering to the principles outlined in the Declaration of Helsinki and approved by the Institutional Ethics Committee (Approval No: 2024/429). The research employed a multi-parameter approach to assess motor performance and ankle mobility in a convenience sample of pre-adolescent male fencers. A priori sample size estimation was conducted using G*Power software (version 3.1.9.3; Heinrich Heine University Düsseldorf, Germany). The calculation was based on the correlation reported by Patti et al. [20], who investigated the relationship between ankle dorsiflexion and vertical jump performance in amateur volleyball players (mean age: 16.5 ± 4.25 years). The correlation coefficient was r = 0.81 (r^2^ = 0.656), which corresponds to a Cohen’s f^2^ of approximately 1.91, indicating an extremely large effect size. A two-tailed correlation model was used with α = 0.05 and statistical power set at 95% (1 − β). The required sample size was calculated as 13; to account for potential dropouts, 15 fencers were recruited.

The measurement process was carried out over a period of two consecutive days in a controlled laboratory environment. On the first day, participants underwent anthropometric assessments, including height, weight, and body fat percentage measurements, as well as a warm-up and familiarization session for biomechanical testing. The second day was dedicated to the execution of dynamic motor performance tasks and biomechanical analyses, ensuring adequate recovery between sessions. Participants were recruited based on predefined inclusion and exclusion criteria. Inclusion criteria comprised being within the pre-adolescent stage of development based on chronological age (9–12 years), confirmed through parent and coach reports, actively participating in fencing training for at least one year, and not having any current or prior chronic or acute injuries that could restrict ankle joint mobility. Exclusion criteria included any diagnosed musculoskeletal disorders or neurological conditions that might interfere with motor performance evaluations.

### 2.2. Body Composition Measurements

The height of the participants was measured using a portable stadiometer (Seca Ltd., Bonn, Germany) with an accuracy of 0.1 cm, ensuring the head was positioned in the Frankfort plane while the body was standing upright with weight evenly distributed on both legs. Body mass and body fat percentage were assessed using a bioelectrical impedance analyzer (Tanita SC-330S, Tokyo, Japan) with a capacity of 270 kg and a sensitivity of 100 g. Body mass index was calculated as weight in kilograms divided by height in metres squared (kg/m^2^). All measurements were conducted in the morning, following an overnight fast, to ensure consistency and minimize the influence of food or fluid intake on body composition data.

### 2.3. Jump Measurement

The counter-movement jump test was conducted to assess the vertical jump performance (cm) of the participants. Vertical jump height was assessed using a contact mat (SmartJump, Fusion Sport, Brisbane, Australia). Participants began in an upright standing position with the trunk straight, hands placed on their hips, feet shoulder-width apart, and knees fully extended (~180°). They were instructed to maintain this position for at least two seconds before initiating a rapid downward movement (push-off phase) until their knees were flexed to approximately 90°, immediately followed by a maximal vertical jump (toe-off phase). During the apex of the jump, participants were instructed to keep their legs extended, and upon landing, to maintain their feet together and knees extended (~180°) [21]. Each participant performed the test three times, and the highest jump height was recorded for analysis. Measurement reliability was confirmed with an intraclass correlation coefficient greater than 90%, ensuring consistent and repeatable results.

### 2.4. Acceleration Measurement

The acceleration of participants was assessed using a fencing-specific lunge test over a 7 m distance. The test distance of 7 m was chosen to reflect the typical range covered during competitive fencing lunges, as previously described in the literature [22]. This test was designed to replicate fencing movements, capturing acceleration during the high-velocity lunge action that is central to the sport. Participants, dressed in full fencing attire, including protective gear and holding a sabre, performed the lunge starting from the en garde position. An electronic dual-beam timing system (Fusion Sport Smart Speed; Fusion Sport, Australia) was used to record the time required to complete the lunge, ensuring high measurement precision. Participants were instructed to perform the movement as explosively and accurately as possible, simulating a competitive bout.

Each participant completed three attempts, with 60 s rest intervals between trials to minimize fatigue effects and ensure consistent performance. The fastest recorded lunge time was used for analysis. To ensure consistency and reliability, all tests were conducted under standardized conditions, and the intraclass correlation coefficient exceeded 0.93, confirming excellent test reliability. Acceleration was calculated using the following formula: “Acceleration (m·s^−2^) = velocity ÷ sprint time”. In this context, velocity was calculated as distance divided by time, and acceleration was derived as a = (d/t)/t = d/t^2^, assuming that the acceleration remained constant throughout the movement.

### 2.5. Ankle Range of Motion Measurements

The range of motion of the ankle joint was measured for dorsiflexion, plantar flexion, eversion, and inversion. All measurements were performed bilaterally using a standardized goniometric method, ensuring consistency and reliability. In fencing-specific posture, one leg is positioned in front (lead leg) and the other remains behind (trailing leg) during lunges. Accordingly, we refer to the ankle of the front foot (lead leg) as the “front foot” and the ankle of the rear foot (trailing leg) as the “rear foot” throughout the manuscript. All participants were right-limb dominant, and for the purpose of data classification, the right leg was consistently defined as the front foot and the left leg as the rear foot in accordance with their typical fencing stance. To ensure measurement accuracy, participants were instructed to avoid compensatory movements, such as internal or external rotation of the hip or excessive knee movement. All measurements were conducted by the same experienced examiner to minimize inter-rater variability. Each movement was measured three times, and the highest value was recorded.

### 2.6. Dorsiflexion and Plantar Flexion

Participants were seated with their knees flexed at 90° to standardize lower limb positioning and reduce gastrocnemius tension. A universal goniometer was aligned with the lateral malleolus as the axis, the stationary arm along the fibula, and the movable arm parallel to the fifth metatarsal. For dorsiflexion, the foot was actively moved toward the tibia, while plantar flexion involved moving the foot downward, away from the tibia [23].

### 2.7. Eversion and Inversion

To assess eversion and inversion, participants were seated with their legs extended and the ankle in a neutral position. The axis of the goniometer was placed over the anterior aspect of the ankle, midway between the malleoli, with the stationary arm aligned with the tibia and the movable arm parallel to the second metatarsal. Participants actively performed eversion (moving the sole outward) and inversion (moving the sole inward) within their maximal range of motion without causing discomfort [24].

### 2.8. Statistical Analysis

Statistical analyses were conducted using the Statistical Package for Social Sciences (version 24; IBM Corporation, Armonk, NY, USA). Data normality was assessed using the Shapiro–Wilk test. Paired sample t-tests were employed to compare rear and front ankle range of motion values. The effect size for this comparison was calculated using Cohen’s d, with values interpreted as small (0.2 ≤ *d* < 0.5), medium (0.5 ≤ *d* < 0.8), and large (*d* ≥ 0.8). The relationships between ankle range of motion, acceleration, and jump height were analyzed using Pearson correlation coefficients, interpreted as follows: trivial (*r* < 0.1), small (0.1 ≤ *r* < 0.3), moderate (0.3 ≤ *r* < 0.5), large (0.5 ≤ *r* < 0.7), very large (0.7 ≤ *r* < 0.9), nearly perfect (*r* ≥ 0.9), and perfect (*r* = 1.0). Before performing the linear regression analysis, the assumptions of linearity, homoscedasticity, and normality of residuals were tested. Linearity and homoscedasticity were assessed using residual scatterplots, while the normality of residuals was evaluated using the Shapiro–Wilk test and Q–Q plots. No violations of these assumptions were detected. Simple linear regression analyses were performed to determine whether ankle range of motion could predict physical performance in acceleration and jump height. All variables were reported as mean ± standard deviation with 95% confidence intervals (CI). Statistical significance was set at an alpha level of *p* < 0.05.

## 3. Results

**Figure 1:**
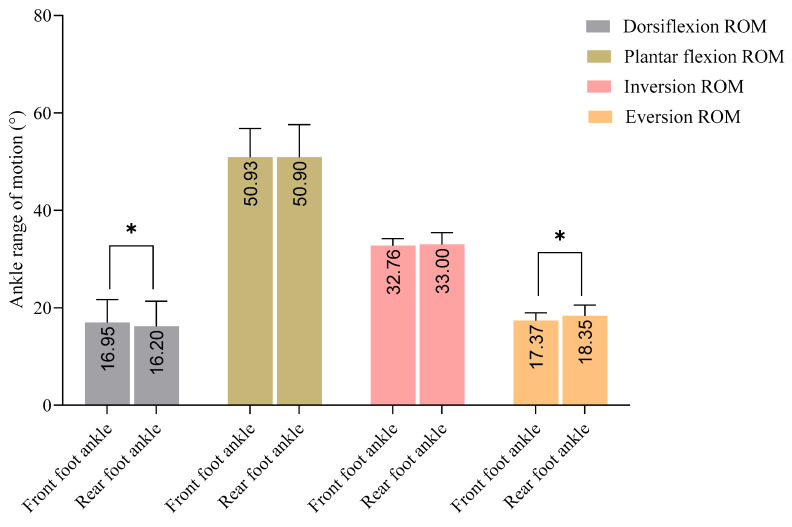
Bilateral comparison of ankle mobility values (front vs. rear foot) in pre-adolescent male fencers. (* = *p* < 0.05).

Participant characteristics for pre-adolescent male fencers are summarized in Table 1. The bilateral comparison revealed significantly greater dorsiflexion in the front foot ankle (*p* = 0.020, *d* = 0.150) and greater eversion in the rear foot ankle (*p* = 0.036, *d* = 0.500), compared to their contralateral counterparts. However, no significant differences were observed in plantar flexion or inversion values (Figure 1).

In Table 2, front and rear foot ankle plantar flexion ROM values demonstrated significant correlations with acceleration (mean ± SD: 1.15 ± 0.31; 95% CI: 0.98, 1.33) and jump height (mean ± SD: 29.32 ± 5.03; 95% CI: 26.53, 32.11) performance variables (*p* < 0.05). Specifically, large correlations were observed between both front and rear foot plantar flexion ROM and acceleration (*p* < 0.05, r = 0.625–0.628, respectively) as well as jump height (*p* < 0.05, r = 0.579–0.647, respectively). However, no significant correlations were identified between bilateral dorsiflexion, inversion, or eversion ROM values and performance metrics (*p* > 0.05).

**Figure 2:**
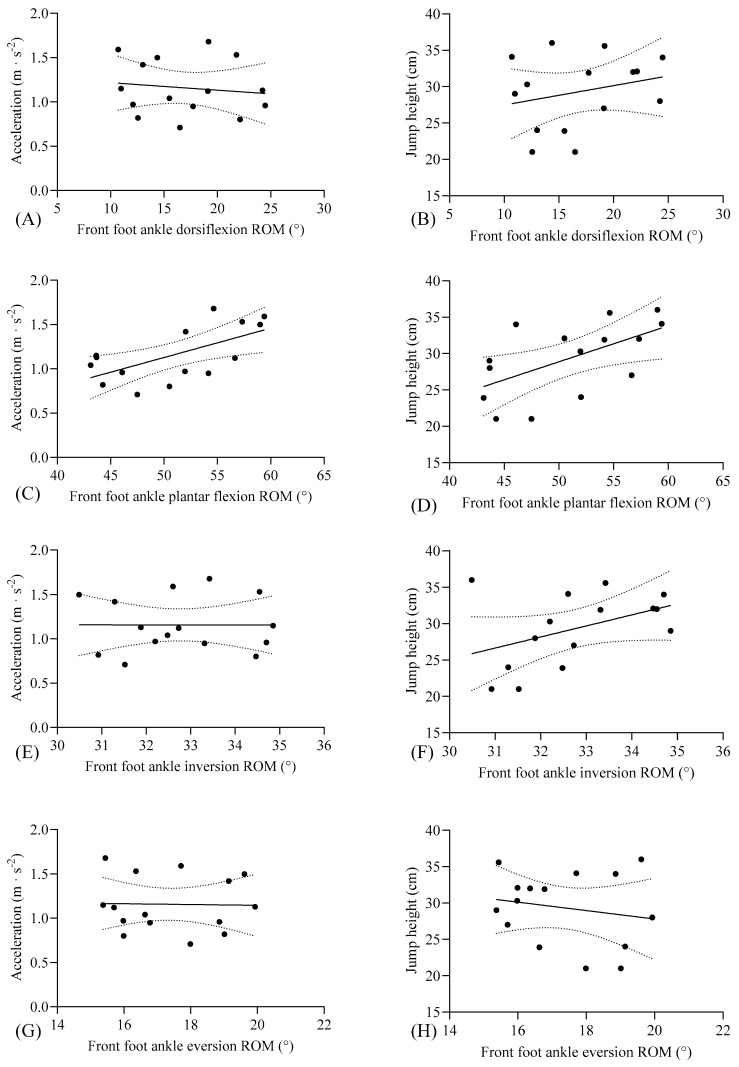
Individual data points, linear regression line (with 95% CI), and correlation between (**A**) front foot ankle dorsiflexion ROM and acceleration, (**B**) front foot ankle dorsiflexion ROM and jump height, (**C**) front foot ankle plantar flexion ROM and acceleration, (**D**) front foot ankle plantar flexion ROM and jump height, (**E**) front foot ankle inversion ROM and acceleration, (**F**) front foot ankle inversion ROM and jump height, (**G**) front foot ankle eversion ROM and acceleration, (**H**) front foot ankle eversion ROM and jump height. Individual data points are presented, as well as a linear regression line with 95% CI. ROM: range of motion.

**Figure 3:**
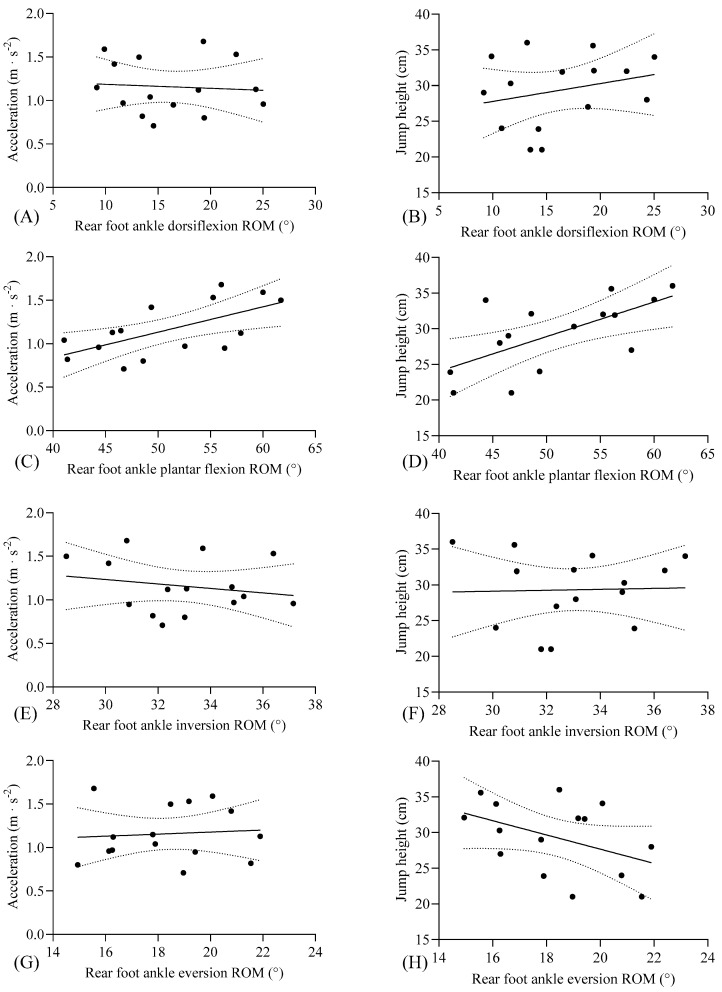
Individual data points, linear regression line (with 95% CI), and correlation between (**A**) rear foot ankle dorsiflexion ROM and acceleration, (**B**) rear foot ankle dorsiflexion ROM and jump height, (**C**) rear foot ankle plantar flexion ROM and acceleration, (**D**) rear foot ankle plantar flexion ROM and jump height, (**E**) rear foot ankle inversion ROM and acceleration, (**F**) rear foot ankle inversion ROM and jump height, (**G**) rear foot ankle eversion ROM and acceleration, and (**H**) rear foot ankle eversion ROM and jump height. Individual data points are presented, as well as a linear regression line with 95% CI. ROM: range of motion.

Front foot plantar flexion ROM emerged as a significant predictor of acceleration (r^2^ = 0.39, *p* < 0.05) and jump height (r^2^ = 0.335, *p* < 0.05), accounting for 33–39% of the variance (Figure 2). Similarly, rear foot plantar flexion ROM significantly predicted acceleration (r^2^ = 0.395, *p* < 0.05) and jump height (r^2^ = 0.418, *p* < 0.05, Figure 3).

## 4. Discussion

The present study explored the relationships between ankle joint mobility and motor performance outcomes in pre-adolescent male fencers. Our findings revealed large correlations between plantar flexion ROM and both acceleration and vertical jump height. These outcomes highlight the functional relevance of the ankle joint in fencing-specific actions, such as lunges and directional changes, which require effective force generation and transfer. The results suggest that distal joint mobility contributes meaningfully to motor performance even at early stages of athletic development and that significant bilateral differences in ankle mobility were observed, likely reflecting functional adaptations to the asymmetrical demands of fencing.

Acceleration and explosive skills are extremely important requirements in branches such as fencing, where the guard is taken against the opponent and back-and-forth moves are frequently applied [25,26]. The relevance of lower-limb power and acceleration capacity in fencing performance is further underscored by prior research comparing athletes across different competitive levels. For instance, previous research has reported significantly greater scores in squat jump, countermovement jump, and long jump among elite fencers compared to national-level athletes, highlighting the discriminative value of explosive strength metrics in fencing contexts [14].

Similarly, peak horizontal velocity and ground reaction force have been shown to be significantly higher in elite compared to intermediate-level fencers (*p* < 0.001 and *p* < 0.01, respectively), reinforcing the role of force production and propulsion in competitive success [15]. Although no statistically significant baseline differences were observed in the standing broad jump between training and control groups in a prior study, a large effect size was reported, suggesting the test’s sensitivity to changes in neuromuscular capacity [22]. Taken together, these findings support the use of short sprint and jump assessments as valid indicators of performance potential in fencing, even in youth athletes.

Sprint acceleration heavily depends on the ankle flexors, which enable rapid ground contact and efficient force transfer [27]. Limited ankle dorsiflexion has been linked to poorer sprinting performance [12]. In a study examining factors affecting lunge speed, a significant correlation was found between rear foot ankle range of motion and horizontal peak velocity (r = 0.550, *p* < 0.05) [15]. During jumping, the optimal plantarflexion range of motion supports a full stretch-shortening cycle in the posterior chain, thereby maximizing vertical force output. Greater dorsiflexion ROM has also been associated with increased knee flexion during landing and reduced ground reaction forces, promoting a posture that lowers anterior cruciate ligament injury risk [28]. Additionally, vertical jump height has been shown to be influenced by ankle mobility (r^2^ = 0.331) [18]. Another study reported a large correlation between active dorsiflexion ROM and countermovement jump performance in young athletes (r = 0.69) [19]. Our findings are consistent with these observations, showing large and statistically significant correlations between both front and rear foot plantar flexion ROM and sprint acceleration (r = 0.625–0.628) and jump height (r = 0.579–0.647).

While the present findings align with studies highlighting the importance of plantar flexion in vertical and horizontal performance [29], the lack of significant correlations between dorsiflexion range of motion and performance outcomes contrasts with earlier research suggesting its relevance in jump mechanics [20]. This discrepancy could be attributed to the specific demands of the tasks evaluated. For example, dorsiflexion is critical during deep squats or reactive jump-like movements that emphasize the eccentric phase [30], whereas the predominantly concentric nature of sprinting and jumping places greater reliance on plantar flexion [31]. These mechanisms likely explain why plantar flexion ROM accounted for 33–42% of the variance in acceleration and jump height, consistent with prior findings that emphasize the role of ankle mobility in rapid movement tasks.

Fencing is a highly dynamic sport characterized by rapid, asymmetric movements, such as lunges, retreats, and sudden changes in direction. This unique biomechanical demand can lead to bilateral asymmetries [32]. The repetitive nature of fencing footwork, with its distinct roles for the front and rear legs, can lead to sport-specific modifications in anatomical and functional differences [33,34]. In one study, the front ankle dorsiflexion torque was found to be 20% stronger than the rear leg on the whole range of motion [35]. Previous research on fencing athletes has shown that these athletes often exhibit enhanced joint mobility and strength imbalances due to the asymmetric loading patterns of their sport [9]. The bilateral differences in dorsiflexion and eversion range of motion observed in this study (small and medium effect sizes) are consistent with the biomechanical requirements of fencing foot movements such as lunges.

This study has certain limitations. While the findings underscore the importance of ankle mobility in acceleration and jump performance, other influential factors such as neuromuscular activation, lower-limb strength, and intersegmental coordination were not examined. The inclusion of only male participants limits the generalizability of the results, as sex-based differences in ankle mobility and joint mechanics have been reported [36]. Additionally, although participants were classified based on chronological age, no direct assessment of biological maturation was conducted. As joint flexibility and range of motion can vary across developmental stages [37], the potential influence of maturation-related differences cannot be ruled out. Moreover, although vertical jump height was assessed using a field-based contact mat, more detailed kinetic information could have been obtained through the use of force platforms. Future studies should consider including female participants, assessing biological maturity, and utilizing dynamic biomechanical tools to enhance ecological validity.

Despite these limitations, this study provides a novel contribution by identifying ankle range of motion as a potential independent parameter in the movement mechanics and classification of fencers. To our knowledge, no prior research has specifically investigated the relationship between ankle range of motion, acceleration, and jump height in this age group, making these findings particularly relevant for advancing the understanding of fencing biomechanics and explosive sports performance.

## 5. Conclusions

This study highlights the critical role of plantar flexion range of motion in predicting short-distance movement speed and jump height in athletic contexts, emphasizing its biomechanical contribution to force generation and propulsion. While dorsiflexion and eversion differences suggest functional asymmetries, their limited impact on performance metrics underscores the primacy of plantar flexion mobility in rapid force-generating movements. These findings contribute to a deeper understanding of ankle joint mechanics in sports performance and support the development of targeted training strategies to optimize athletic outcomes.

## Figures and Tables

**Table 1:** Descriptive statistics for the participant characteristics in pre-adolescent male fencers (*n* = 15).

Variables	Mean ± Standard Deviation	95% CI
Age (years)	10.86 ± 0.91	10.35, 11.37
Height (m)	1.51 ± 0.08	1.47, 1.56
Weight (kg)	41.91 ± 5.52	38.85, 44.97
Body mass index (kg·m^−2^)	18.18 ± 1.81	17.18, 19.19
Body fat percentage (%)	12.43 ± 2.90	10.73, 13.95

**Table 2:** Pearson correlation coefficients between front and rear foot ankle mobility values and motor performance scores in pre-adolescent male fencers (*n* = 15).

Correlation Matrix	Front		Rear	
Acceleration (m·s^−2^)	*r* value	*p* value	*r* value	*p* value
Dorsiflexion ROM	−0.127	0.622	−0.076	0.789
Plantar flexion ROM	0.625	0.013	0.628	0.012
Inversion ROM	0.001	0.997	−0.199	0.477
Eversion ROM	−0.023	0.936	0.083	0.769
Jump height (cm)				
Dorsiflexion ROM	0.250	0.368	0.257	0.356
Plantar flexion ROM	0.579	0.024	0.647	0.009
Inversion ROM	0.431	0.109	0.032	0.911
Eversion ROM	−0.185	0.508	−0.435	0.105

## Data Availability

The data that support the findings of this study are available from the corresponding author upon reasonable request.

## Abbreviations

The following abbreviations are used in this manuscript:

CMJ	Counter-movement jump
ROM	Range of motion
